# Interferon Gamma Induces the Increase of Cell-Surface Markers (CD80/86, CD83 and MHC-II) in Splenocytes From Atlantic Salmon

**DOI:** 10.3389/fimmu.2021.666356

**Published:** 2021-05-13

**Authors:** Byron Morales-Lange, Felipe Ramírez-Cepeda, Paulina Schmitt, Fanny Guzmán, Leidy Lagos, Margareth Øverland, Valentina Wong-Benito, Mónica Imarai, Derie Fuentes, Sebastián Boltaña, Javier Alcaíno, Carlos Soto, Luis Mercado

**Affiliations:** ^1^ Grupo de Marcadores Inmunológicos en Organismos Acuáticos, Instituto de Biología, Pontificia Universidad Católica de Valparaíso, Valparaíso, Chile; ^2^ Laboratorio de Síntesis de Péptidos, Núcleo Biotecnología de Curauma, Pontificia Universidad Católica de Valparaíso, Valparaíso, Chile; ^3^ Department of Animal and Aquaculture Sciences, Faculty of Biosciences, Norwegian University of Life Sciences, Ås, Norway; ^4^ Laboratorio de Inmunología, Departamento de Biología, Centro de Biotecnología Acuícola, Universidad de Santiago de Chile, Santiago, Chile; ^5^ Aquaculture and Marine Ecosystems, Center for Systems Biotechnology, Fraunhofer Chile Research, Santiago, Chile; ^6^ Department of Oceanography, University of Concepción, Concepción, Chile; ^7^ AquaAdvise—Fundación Chile, Puerto Montt, Chile; ^8^ Salmones Camanchaca, Puerto Montt, Chile

**Keywords:** *Salmo salar*, fish interferon-gamma, fish dendritic cells, fish splenocytes, fish phenotypic markers, fish zbtb46

## Abstract

Type II interferon gamma (IFNγ) is a pleiotropic cytokine capable of modulating the innate and adaptive immune responses which has been widely characterized in several teleost families. In fish, IFNγ stimulates the expression of cytokines and chemokines associated with the pro-inflammatory response and enhances the production of nitrogen and oxygen reactive species in phagocytic cells. This work studied the effect of IFNγ on the expression of cell-surface markers on splenocytes of Atlantic salmon (*Salmo salar*). *In vitro* results showed that subpopulations of mononuclear splenocytes cultured for 15 days were capable of increasing gene expression and protein availability of cell-surface markers such as CD80/86, CD83 and MHC II, after being stimulated with recombinant IFNγ. These results were observed for subpopulations with characteristics associated with monocytes (51%), and features that could be related to lymphocytes (46.3%). In addition, a decrease in the expression of *zbtb46* was detected in IFNγ-stimulated splenocytes. Finally, the expression of IFNγ and cell-surface markers was assessed in Atlantic salmon under field conditions. *In vivo* results showed that the expression of *ifnγ* increased simultaneously with the up-regulation of *cd80/86*, *cd83* and *mhcii* during a natural outbreak of *Piscirickettsia salmonis*. Overall, the results obtained in this study allow us to propose IFNγ as a candidate molecule to stimulate the phenotypic progression of a small population of immune cells, which will increase antigen presenting cells markers. Thereby, modulatory strategies using IFNγ may generate a robust and coordinated immune response in fish against pathogens that affect aquaculture.

## Introduction

Interferon gamma (IFNγ) is a soluble pleiotropic cytokine member of type II class of interferons (1). This molecule has been described in many groups of vertebrates, such as chondrichthyes, jawed fish, amphibians, birds and mammals, suggesting that IFNγ appeared at least 450 million years ago, before the divergence between fish and tetrapods (2).

In higher vertebrates, IFNγ is mostly expressed and secreted by natural killer cells (NK) and T helper type 1 cells (Th1) and it acts over different cell types, including macrophages (Mф), dendritic cells (DCs) and T cells, regulating both innate and adaptive immune responses through the activation of JAK-STAT pathway (3). In mammals, stimulation of cells with IFNγ has been shown to modulate the expression of cell surface markers. Specifically, the stimulation of DCs with IFNγ causes an increase in the transcriptional expression of CD40, CD80, CD86 and the secretion of IL-12, which can subsequently induce the activation of CD4^+^ and CD8^+^ T cells (4). Moreover, IFNγ can positively regulate the expression of Major Histocompatibility Complex class II (MHC II) (3).

In teleost fish, IFNγ has been characterized in different families such as cichlids, cyprinids and salmonids (5). In these animals, IFNγ is a molecule capable of improving phagocytosis and enhancing the nitric oxide responses of phagocytes by the modulation of cytokines expression, e.g. tumor necrosis factor-alpha (TNFα), interleukin 1 beta (IL-1β), interleukin 6 (IL-6) and IL-12 (6). In addition, IFNγ is also capable of modulating the antiviral immune system by activating molecules such as Mx, ISG15 and RSAD2 (5). Furthermore, IFNγ-induced MHC II expression signaling pathways (7) in grass carp, and in rainbow trout, IFNγ decreases the expression of Zinc Finger and BTB Domain Containing 46 (ZBTB46) in primary cultures of head kidney and RTS-11 cell line (8). ZBTB46 is a transcriptional factor acting as a global repressor of the maturity of Antigen Presenting Cells (APCs), controlling the expression of cell-surface markers (9).

The detection and analysis of cell-surface markers using molecular and phenotypic strategies allows the characterization and function of the dendritic cells in fish (10). Examples of these markers are CD80/86, CD83 and MHC II.

CD80/86 is a type I membrane glycoprotein expressed on the surface of APCs, which promotes a co-stimulatory signal to activate a T cell response (10). In fish, CD80/86 shares sequence similarity and a phylogenetic relationship with mammalian CD80 and CD86 genes (11). In higher vertebrates, CD80 and CD86 are encoded by different sequences (CD80: B7-1 and CD86: also named B7-2). However, in salmonids, such as trout, it is encoded in CD80/86A and CD80/86B., suggesting that CD80 and CD86 arose by gene duplication in the tetrapod branch, after the separation of fish (12).

CD83 is another important cell-surface marker. This molecule is an integral membrane protein that belongs to the immunoglobulin superfamily and it acts as an immuno-regulator protein, inducing immune responses mediated by T cells and delivering costimulatory signals, which activate naive T lymphocytes and stimulate their proliferation (13, 14).

Furthermore, MHC II is a cell-surface marker formed by alpha and beta chains, each of them composed by two domains. MHC II resides on the surface of APCs and presents peptides derived from exogenously proteins to CD4+ T-cells (15). In salmonids, it has been reported that IFNγ can modulate MHC II, increasing its gene expression level (6).

To better understand the immune system of nonclassical biological models, more knowledge about the identification cell-surface markers is required. In this context, Atlantic salmon (*Salmo salar*) is a suitable candidate due to its economic importance in aquaculture (16), and the fact that intensive farming system increases the occurrence of pathogenic diseases in fish (17). This later result makes the host’s immune response play a key role in the animal’s survival and the sustainability of the production process.

In the present work, the effect of recombinant IFNγ on the expression of CD80/86, CD83 and MHC II was evaluated in splenocytes of Atlantic salmon at the transcriptional and protein level. Furthermore, the expression of IFNγ and cell-surface markers were assessed under field conditions. This study suggests that IFNγ stimulates the phenotypic progression of a small subpopulation of splenocytes, by increasing the levels of cell-surface markers. These findings may contribute to the understanding of the immune response in *S. salar* and propose the use of IFNγ as an immuno-modulator in fish to generate a robust and coordinated response against pathogens that affect aquaculture.

## Materials and Methods

### Isolation of Splenocytes From Atlantic Salmon and Induction With rIFNγ

Twenty healthy fish of 300 ± 30 g were obtained from NeoSalmon fish farm (Los Lagos region, Chile). Each fish’s spleen was removed under aseptic conditions. All organs were homogenized on a cell strainer (100 μm^2^) with Leibovitz`s L-15 medium (Gibco). Thereafter, 1 ml of each cell suspension was added to a Percoll gradient (2 ml 34%—2 ml 51%). The gradients were centrifuged at 800×*g* for 40 min at 18°C. Then, the mononuclear fraction of cells which have sedimented to the interface from each gradient were recovered by carefully pipetting, washed with 15 ml of L-15 medium and centrifuged at 180×*g* for 7 min at 18°C. The supernatant was removed and the cells were resuspended in 1 ml of L-15 medium. Cell count and viability test were performed by the Trypan blue method. Later, the cells from each fish were seeded in four wells (12-well plates) at 1 × 10^6^ cells ml^−1^ in complete L-15 medium (10% FBS and 1% penicillin–streptomycin) and stabilized for 2 h at 20°C following the method described by Bassity and Clark (18). Finally, non-adherent cells were removed to select mononuclear adherent cells. Fresh complete L-15 medium was added (2 ml per well) in adherent cells for overnight incubation. The next day, the wells with cells were divided into control and induced groups. To control splenocytes, 50 µl of sterile phosphate buffer saline (PBS) was added, while cells from induced group were stimulated with recombinant IFNγ from *S. salar* (Ss rIFNγ) (19) at 100 ng ml^−1^ (20) for 8 or 15 days. The L-15 medium was not changed during the cell incubation time. The cells were observed daily by optical microscopy and the color of the medium was checked daily as an indicator of any pH variation.

The splenocytes distribution for each assay (RT-qPCR, western blot, immunofluorescence, and flow cytometry) is described in **Table 1**. To establish morphological parameters in splenocytes, samples were obtained from four cultures without inducers in days 1 and 15. Then, Giemsa stain and flow cytometry was performed. For flow cytometry, cells were recovered in IF medium (2% FBS in PBS) and centrifuged at 180×*g* for 7 min at 18°C. Then, cells were resuspended in 1 ml of IF medium and incubated with Live/Dead (MERCK) diluted 1:1,000 for 10 min in the dark. Later, cells were washed twice with IF medium and fixed with 4% paraformaldehyde for 10 min at room temperature. Finally, cells were washed and resuspended on IF. The results were obtained using a Gallios Flow Cytometer (Beckman Coulter).

**Table 1:** Splenocytes distribution by assay.

	Splenocytes per fish			
	Well 1	Well 2	Well 3	Well 4
**Fish1**	qPCR_Control_8d	qPCR_Control_8d	qPCR_IFNγ_8d	qPCR_IFNγ_8d
**Fish2**	qPCR_Control_8d	qPCR_Control_8d	qPCR_IFNγ_8d	qPCR_IFNγ_8d
**Fish3**	qPCR_Control_8d	qPCR_Control_8d	qPCR_IFNγ_8d	qPCR_IFNγ_8d
**Fish4**	qPCR_Control_8d	qPCR_Control_8d	qPCR_IFNγ_8d	qPCR_IFNγ_8d
**Fish5**	qPCR_Control_15d	qPCR_Control_15d	qPCR_IFNγ_15d	qPCR_IFNγ_15d
**Fish6**	qPCR_Control_15d	qPCR_Control_15d	qPCR_IFNγ_15d	qPCR_IFNγ_15d
**Fish7**	qPCR_Control_15d	qPCR_Control_15d	qPCR_IFNγ_15d	qPCR_IFNγ_15d
**Fish8**	qPCR_Control_15d	qPCR_Control_15d	qPCR_IFNγ_15d	qPCR_IFNγ_15d
**Fish9**	WB_Control_8d	IFAT_Control_8d	WB_IFNγ_8d	IFAT_IFNγ_8d
**Fish10**	WB_Control_8d	IFAT_Control_8d	WB_IFNγ_8d	IFAT_IFNγ_8d
**Fish11**	WB_Control_8d	IFAT_Control_8d	WB_IFNγ_8d	IFAT_IFNγ_8d
**Fish12**	WB_Control_8d	IFAT_Control_8d	WB_IFNγ_8d	IFAT_IFNγ_8d
**Fish13**	WB_Control_15d	IFAT_Control_15d	WB_IFNγ_15d	IFAT_IFNγ_15d
**Fish14**	WB_Control_15d	IFAT_Control_15d	WB_IFNγ_15d	IFAT_IFNγ_15d
**Fish15**	WB_Control_15d	IFAT_Control_15d	WB_IFNγ_15d	IFAT_IFNγ_15d
**Fish16**	WB_Control_15d	IFAT_Control_15d	WB_IFNγ_15d	IFAT_IFNγ_15d
**Fish17**	Flow_Control_15d	Flow_Control_15d	Flow_IFNγ_15d	Flow_IFNγ_15d
**Fish18**	Flow_Control_15d	Flow_Control_15d	Flow_IFNγ_15d	Flow_IFNγ_15d
**Fish19**	Flow_Control_15d	Flow_Control_15d	Flow_IFNγ_15d	Flow_IFNγ_15d
**Fish20**	Flow_Control_15d	Flow_Control_15d	Flow_IFNγ_15d	Flow_IFNγ_15d

Control: splenocytes without induction. IFNγ: splenocytes induces with Ss IFNγ. 8d and 15d: days of incubation. Assays: qPCR (RT-qPCR), WB, western blot; IFAT, Immunofluorescence; Flow, flow cytometry.

### *Piscirickettsia salmonis* Outbreaks Surveillance in Commercial Fish Farms

During the five months between June and October of 2018, a pathogen outbreak surveillance was carried out in two commercial fish farms (FarmA: Punta Islotes and FarmB: Puelche), property of Salmones Camanchacha SA. Both fish farms were located in Los Lagos Region, Chile (FarmA: 42°55**’**08.3**”**S 72°46**’**23.9**”**W and FarmB: 41°44**’**30.0**”**S 72°39**’**42.0**”**W).

Fifty adult fish at the final stage of production and not related to mortality events were collected per month, from each farm. All fish were euthanized by anesthetic overdose (BZ**^®^**-20, Veterquimica) and samples from spleen were taken and then stored in RNAlater**^®^** at −80**°**C until processing. From the fifty fish collected each month, on each farm, fifty spleens were subjected to RNA extraction, and then 10 samples were pooled to obtain cDNA.

FarmA did not report any pathogenic outbreaks along the surveillance period. On the contrary, FarmB reported two natural outbreaks of *P. salmonis* in August and October. The presence of *P. salmonis* in Atlantic salmon was determined by RT-qPCR in head kidney samples according to Flores-Herrera et al. (21). The detection of other salmonids pathogens, such as *Renibacterium salmoninarum*, infectious salmon anemia virus and infectious pancreatic necrosis virus was also carried out by RT-qPCR in head kidney samples. The detection of these pathogens was negative in all fish.

### RNA Extraction and Quantitative Analysis of Immune Gene Expression by RT-qPCR

Quantitative analysis of gene expression was determined according to Álvarez et al. (22). Briefly, RNA extraction was performed using Total RNA Kit I (E.Z.N.A.) following the instructions from the manufacturer. Then, total RNA was quantified using a NanoDrop spectrophotometer (NanoDrop Technologies) and cDNA synthesis was carried out using 200 ng total RNA, 50 ng oligo-(dT)12-18 (Thermo Scientific), 1 mM dNTPs (Promega), 1 U Rnasin (Promega) and 200 U M-MLV reverse transcriptase in reverse transcriptase buffer (Promega) following the manufacturer**’**s protocol. The protocol described above was used to obtain RNA from samples from both *in vitro* splenocytes and outbreak surveillance in fish farms. Additionally, after cDNA synthesis, samples were grouped into five pools of ten animals each (cDNA per fish 1:1), per month, from each farm, separately.

Quantitative PCR (qPCR) reactions were performed in triplicate and conducted in an Mx3000P qPCR System (Agilent Technologies) for all samples. Each reaction consisted in a total amount of 15 µl containing Brilliant II SYBR**^®^** Green QPCR MM (Agilent Technologies), 0.2 µM of each primer, and 12 ng of cDNA diluted in sterile ultra-pure water. The RT-qPCR conditions were: denaturation step of 3 min at 94**°**C before a total of 35 cycles of 94**°**C for 15 s, annealing at 55**°**C for 15 s, extension time at 72**°**C for 15 s. and melting curve (95**°**C for 15 s, 25**°**C for 1 s, 70**°**C for 15 s and 95**°**C for 1 s). Specific primers were designed to amplify *cd80/86*, *cd83*, *mhc-ii*, *zbtb46*, *ifnγ* and *elongation factor 1* (*ef-1*) was chosen as reference gene for normalization (**Table 2**). Primer pair efficiencies (E) were calculated from six serial dilutions of pooled cDNA for each primer pair according to the equation: E = 10^[−1/slope]^. Relative expression was calculated using the 2^−ΔΔCt^ Method (23).

**Table 2:** List of primers used for gene amplification by quantitative PCR.

Gene	Primer	Sequence	Tm	Reference
cd80/86	Forward	GATGTTGAGTGGAGCCTGAA	55°C	GenBank: CAQ51440.1 NCBI Gene ID: 106613207
	Reverse	GACGACAGAGAACAGCATAGAG	55°C	
mhc ii	Forward	AATCAGAGTGACCTGGTTGAG	55°C	GenBank: CAD27720.1 NCBI Gene ID: 106565699
	Reverse	GTGGGAGAGGATCTGGTAGTA	55°C	
cd83	Forward	AGGATTCGGTTCTGAGATGTAAAG	55°C	GenBank: ABC68619.1 NCBI Gene ID: 100136479
	Reverse	GTGACAGCCTCTTCATCAGTAG	55°C	
zbtb46	Forward	CCTCTACTGCCAGGTTAAGAAAG	55°C	NCBI Reference Sequence: XM_014134725.1
	Reverse	CAGAGTACATGAAGTCCAGGATG	55°C	
ifnγ	Forward	ATGCTGCTCAGTTCACATCA	61°C	NCBI Reference Sequence: NM_001171804.1
	Reverse	ACGTCCAGAACCACACTCAT	61°C	

### Production and Validation of Antibodies

Polyclonal antibodies against synthetic antigenic peptides from Atlantic salmon were obtained in mouse CF-1 and New Zealand rabbit (**Table 3**). Each peptide was selected and chemically synthesized using a Fmoc strategy according to Rojas et al. (24), considering parameters of antigenicity, hydrophobicity, accessibility, flexibility, and 3D modeling by Phyre2 (25). UniProt database (UniProt consortium) was used to obtain the amino acid sequences for each marker (CD80/86: W0U058, CD83: Q27YA6 and MHCII alpha chain: Q5ZQM6). For the final selection of antigenic peptides, only the extracellular region of each protein (predicted by Phobius EMBL-EBI) was considered (**Figure 3A**).

**Table 3:** Primary antibodies against cell-surface markers of Atlantic salmon.

Molecule	Antibody production	Peptide Sequence	UniProt Database
CD80/86	Mouse	TCSSDNGYPRRDVEW	W0U058
MHC-II	Mouse	PPHSSIYPRDDVDLG—TLICHVGFHPAPVR	Q5ZQM6
CD83	Rabbit	CAVDSGRYKCLLAAPVC	Q27YA6

For antibody production, animals were subcutaneously injected at 1, 14 and 28 days with 150 μg (for mouse) and 300 μg (for rabbit) of the antigenic peptide diluted 1:1 with FIS peptide (FISEAIIHVLHSR), a T helper cell activator (26), and 1:1 in Freund**’**s adjuvant (Thermo). The antiserum was collected on day 45, centrifuged at 500×*g* for 15 min at 4**°**C and the supernatant was stored at −20**°**C. The antibodies were purified with NAb Protein G Spin Columns (Thermo) following the supplier’s instructions. The antibody affinity was determined by indirect ELISA against a serial dilution of the synthetic peptide, while the antibody specificity was determined by western blotting (27), using a biological sample of a pool from four spleens. The specificity of the antibodies against CD80/86, CD83 and MHCII has been previously reported by Nombela et al. (28).

### Protein Detection

Western blotting was performed to detect cell-surface markers in protein samples from control and Ss rIFNγ-induced splenocytes at 8 and 15 days, according to Schmitt et al. (29). The membranes were incubated for 60 min at room temperature with the antibody anti-synthetic epitope (CD80/86 1:200, CD83 1:100 and MHC-II 1:200), followed by three washing with PBS-tween. A secondary antibody (anti-IgG-HRP, Thermo) was used diluted 1:5,000 in 1% BSA for 60 min at room temperature. Specific bands were visualized by chemiluminescence with the Westar Supernova kit (Cyanagen).

Immunofluorescence analyses were carried out using control and Ss rIFNγ-induced splenocytes after 15 days of culture. Briefly, cells were fixed with 4% paraformaldehyde in PBS for 15 min, washed and centrifuged at 180×*g* for 7 min at 4**°**C. After, cells were adhered to a glass slide pre-treated with poly-lysine by centrifugation in a cytospin (Hettich Universal 32R) at 180×*g* for 10 min. Blocking was performed with 5% BSA for 30 min and then the slides were washed with 0.2% PBS-tween. To avoid auto-fluorescence, the slides were incubated in ammonium chloride (50 mM) for 10 min. Thereafter, the slides were incubated for 60 min at room temperature with the antibody anti-synthetic epitope (using the same dilutions described above). Followed by three washes with PBS-tween, a secondary antibody was used diluted 1:200 (in 1% BSA) for 60 min in the dark at room temperature (Alexa 568 goat anti-mouse and Alexa 635 goat anti-rabbit). Finally, the slides were incubated with Syto 9 for 5 s (1:1,000) and mounted in Vectashield Mounting Medium (Vector Lab). Microscope images were taken using a 40 × 1.25 Oil HCX PL APO CS lens, Leica TCS SP5 II spectral confocal microscope (Leica Microsystems).

For the detection of APCs markers in 15-day splenocytes by flow cytometry, we followed the protocol described in *Isolation of Splenocytes From Atlantic Salmon and Induction With rIFNγ*. In addition, cells were labelled with primary antibodies for cell markers: CD80/86 (1:200), CD83 (1:100) and MHC II (1:200) for 1 h at room temperature. After several washes, cells were incubated with the respective secondary antibody: anti-mouse IgG-Alexa 488 and anti-rabbit IgG-Alexa 647 for 1 h at room temperature and washed again with IF medium. Finally, cells were fixed with 4% paraformaldehyde and resuspended in IF at room temperature in dark. The results were obtained in a Gallios Flow Cytometer (Beckman Coulter).

### Data Analysis

For RT-qPCR data, GraphPad v7.03 was used to calculate estimated means, standard deviation, Student**’**s t-test (two-tailed), one-way ANOVA and Tukey test for multiple comparisons. Correlations were performed using corrplot package in R (30). Differences were considered significant when the *P*-value was **<**0.05. Flow cytometry results were analyzed using FlowJo vX.

## Results

### Morphological Characterization of Mononuclear Splenocytes From Atlantic Salmon

**Figure 1:**
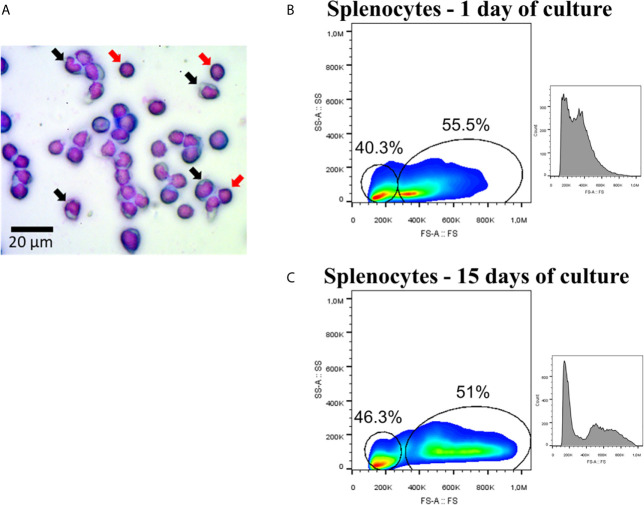
Splenocytes of Atlantic salmon. **(A)** Mononuclear fraction. Black arrows showed cells with morphology associated with monocytes, while red arrows showed examples of cells with lymphocyte-associated morphology. The image was taken with magnification 400×. **(B)** Flow cytometry (FS-A/SSC-A and histogram/FS-A) using splenocytes at 1 day of culture. **(C)** Flow cytometry (FS-A/SSC-A and histogram/FS-A) using splenocytes at 15 day of culture.

The mononuclear fraction of splenocytes from Atlantic salmon cultured for up to 15 days did not show any significant differences in cell viability between 1-day (97.4%) and 15-day (95.6%) cultures. Splenocytes displayed characteristics associated with two types of mononuclear morphology when analyzed under optical microscopy: one subpopulation with nuclear features similar to monocytes (**Figure 1A**, black arrows), and other subpopulation with a circular nucleus that occupies a large portion of the cellular space, similar to lymphoid cells (**Figure 1A**, red arrows). Flow cytometry analysis showed that the percentages of these type of cells were 40.3 and 55.5% respectively, in samples from 1 day of culture (**Figure 1B**), and 46.3 and 51% respectively at the 15-day cultures (**Figure 1C**). Moreover, it was possible to establish that splenocytes cultured for 15 days increased in their size (FS-A) compared to those cultured for 1 day (**Figures 1B, C**).

### Gene Expression of Cell-Surface Markers in Splenocytes of Atlantic Salmon

**Figure 2:**
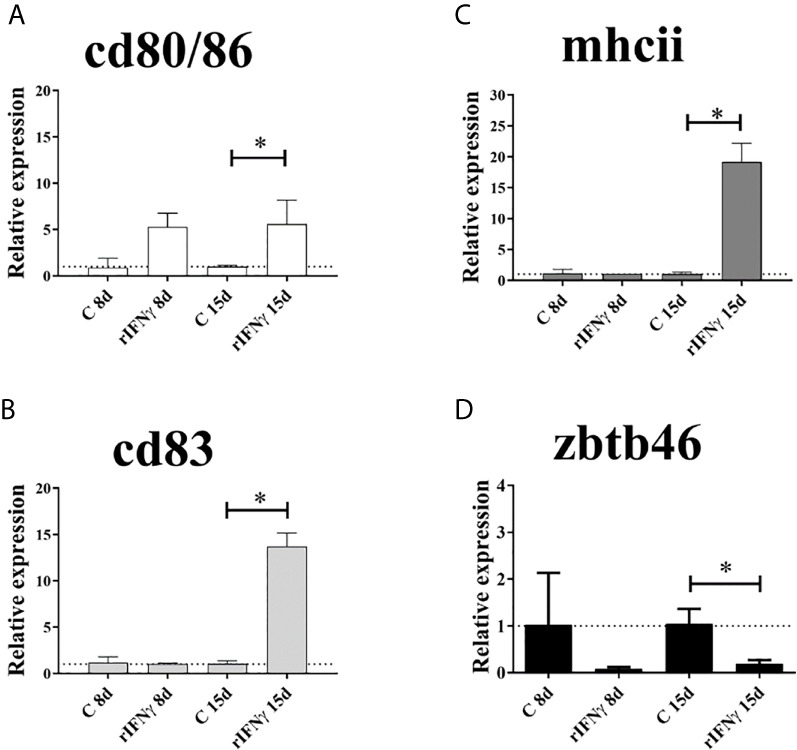
Gene expression of cell-surface markers by RT-qPCR in splenocytes of Atlantic salmon cultured 8 and 15 days (with and without induction of IFNγ). **(A)** *cd80/86*, **(B)** *cd83*, **(C)** *mhcii* and **(D)** *zbtb46*. The data were plotted in bars (n = splenocytes from four fish) showing the relative expression in fold change related to the control group. *: Significant difference (*P < *0.05) by Student**’**s t-test two-tailed.

The analysis of gene expression assessed by RT-qPCR showed a significantly increase of cell-surface markers *cd80/86*, *mhcii* and *cd83* in splenocytes induced with Ss rIFNγ for 15 days, compared to the control group without induction (*P* ***<***0.05) (**Figures 2A–C**). Specifically, *cd80/86* displayed a 5.6-fold increase in treated splenocytes (**Figure 2A**); *mhcii* exhibited 19.15-fold increase compared to control cells (**Figure 2B**) and *cd83* showed 13.73-fold expression increase in induced splenocytes (**Figure 2C**). Regarding to *zbtb46*, this molecule showed a significant decrease of 5-fold decreased after 15-days stimulation with Ss rIFNγ, compared to control splenocytes (**Figure 2D**).

### Production and Validation of Antibodies

**Figure 3:**
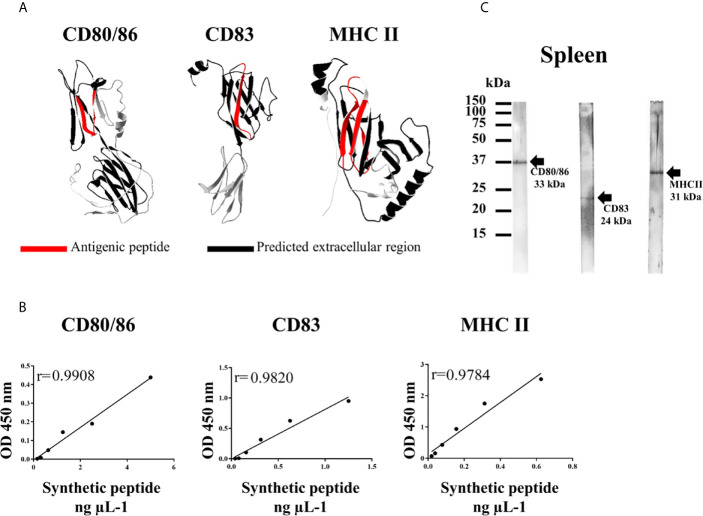
Production and validation of antibodies against cell-surface markers of Atlantic salmon. **(A)** Three-dimensional modeling by homology for each protein, on the left: CD80/86, in the center: CD83 and on the right: MHC II. In red: antigenic peptides selected for synthesis and immunization. In black: the predicted extracellular region. **(B)** Calibration curve by indirect ELISA to establish the proportional relationship between the detection of the antibody produced (at 450 nm) and the concentration of the synthetic peptide (ng μl^−1^). r: correlation coefficient. **(C)** Western blot to determinate the specificity of each antibody in a sample of spleen proteins (pool of four fish).

The analysis to select candidate peptides (**Table 3**) showed that the peptide for CD80/86 had fifteen amino acids with a molecular weight of 1,784.88 Da (**Figure 3A**, left panel). For CD83, the peptide was an epitope of thirteen amino acids and 1,392.64 Da (**Figure 3A**, central panel), and finally for MHC II, the peptide was a combination of two antigenic epitopes with a molecular weight of 3,196.63 Da (**Figure 3A**, right panel).

After the production of antibodies against the cell-markers of *S. salar*, the affinity ratio of the purified antibodies was determined against the corresponding synthetic peptide by indirect ELISA (**Figure 3B**). The ability of each antibody to recognize the antigen was demonstrated as a linear behaviour against the synthetic epitope used. In addition, a direct and proportional relation between the detection of the antibody and concentration of the synthetic peptide (ng μl^−1^) was determined by the correlation coefficient (r) (CD80/86: r = 0.9816; MHCII: r = 0.9953 and CD83: r = 0.9908). The pre-immune sera from each animal did not show any detection for the different antigens.

The specificity of the produced antibodies against the native proteins was determined by western blotting using protein extracts from Atlantic salmon spleen samples (**Figure 3C**). Results showed that each antibody detected single band proteins at the expected molecular weight for each cell-surface marker: 33 kDa for CD80/86, 31 kDa for MHC IIα, and 24 kDa for CD83.

### Detection of Cell-Surface Markers in IFNγ-Induced Splenocytes at Protein Level

**Figure 4:**
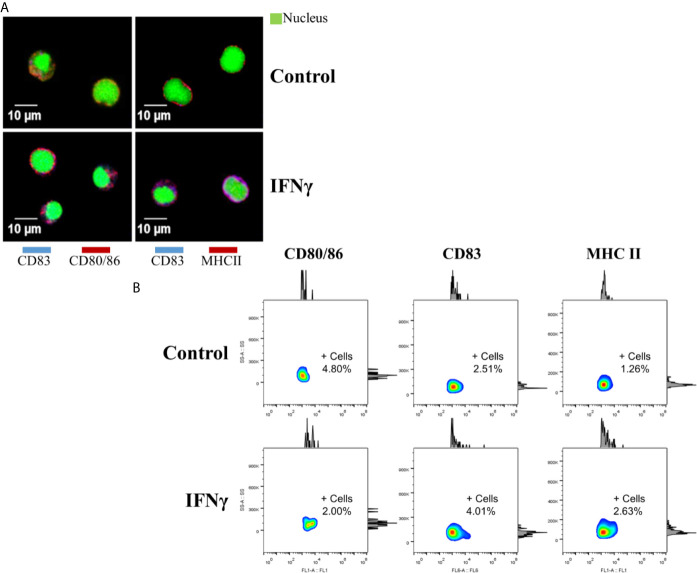
Detection of cell-surface markers at the protein level. **(A)** Confocal microscopy of cell-surface markers in splenocytes of Atlantic salmon cultured for 15 days. Top panels (left and right), cells without IFNγ (control). Lower panels (left and right), cells with IFNγ as inducer. Left panels: in red, CD80/86. Right panels: in red, MHCII. CD83 shown in blue, in all panels. In green (in all panels) Syto9 as a nuclear contrast. Images were taken using 800x amplification. **(B)** Flow cytometry in splenocytes cultured for 15 days (Side Scatter-Area versus fluorescence). Top panels: Control group without inducer. Lower panels: IFNg group. +Cells: Percentage of positive cells for the corresponding marker.

To evaluate the effect of IFNγ on mononuclear splenocytes after 15 days of culture, the detection of the three cell-surface markers was performed by Immunofluorescence and confocal microscopy (**Figure 4A**). These results showed a positive labeling of CD80/86 and MHC II in cells with a diameter close to 10 µm (similar to monocytes), from both IFNγ-induced and control splenocytes. However, the co-localization of CD83 with CD80/86 and MHC II was found more recurrently in IFNγ-induced splenocytes (81.8 and 91.6%, respectively), compared to 33 and 60% from the control group.

In parallel, specific bands on the expected molecular weight for the three cell-surface markers were detected in both IFNγ-induced and control splenocytes by western blotting (**Supplementary Figure 1**). Still, CD83 band intensity was higher in splenocytes induced with IFNγ for 15 days.

Consistently, an increase in the mean fluorescent intensity (MFI) of the three cell-surface markers, expressed as a fold change ratio between IFNγ-induced and control splenocytes was detected by flow cytometry (**Figure 4B**). CD80/86 showed 1.87-fold, CD83: 2.25-fold and MHC II: 1.33-fold. Finally, a low percentage of positive cells was detected for each marker; CD80/86: 4.80% (control) and 2.00% (IFNγ), CD83: 2.51% (control) and 4.01% (IFNγ), and MHC II: 1.26% (control) and 2.63% (IFNγ).

### Gene Expression of *ifnγ, cd80/86, cd83* and *mhcii* in Spleen of Atlantic Salmon During *Piscirickettsia salmonis* Outbreaks Surveillance

The gene expression of the *ifnγ, cd80/86, cd83* and *mhcii* was assessed in Atlantic salmon spleen from two fish farms (FarmA and FarmB) during a 5-month outbreak surveillance. During this time, no cases of *P. salmonis* or other pathogens were reported from FarmA. Nevertheless, two natural outbreaks of *P. salmonis* were reported in FarmB (on August and October).

**Figure 5:**
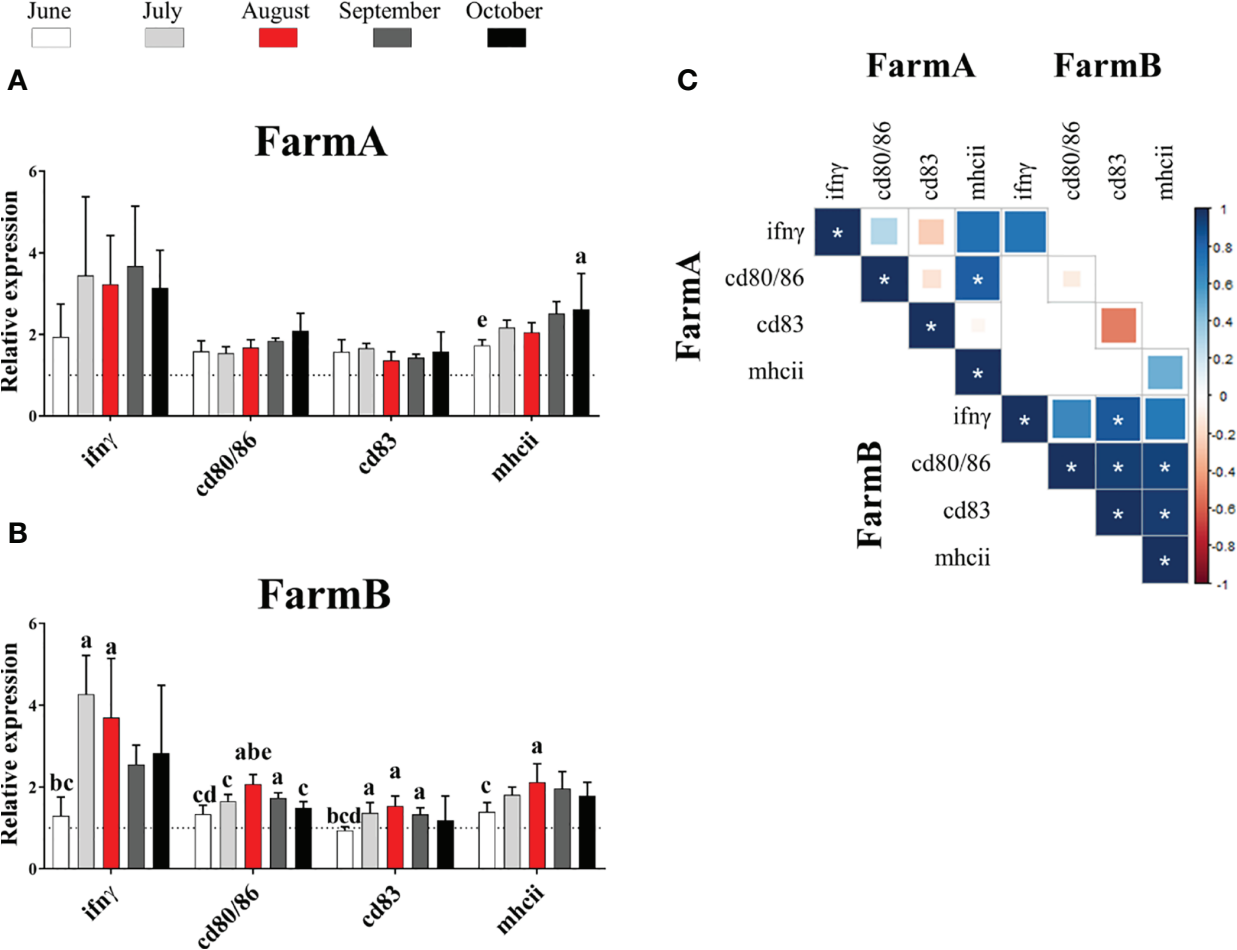
Gene expression of *ifnγ* and cell-surface markers (*cd80/86*, *cd83* and *mhcii*) in the spleen of fish from two fish farms. FarmA **(A)** and FarmB **(B)** during the time period June–October 2018. The data were plotted in bars (n = five pools, 10 fish per pool) using relative expression in fold of change. Lowercase letters (a, b, c, d and e): significant differences (*P* ***< ***0.05) compared to June, July, August, September and October, respectively by Tukey**’**s multiple comparison test. In white: June. In light gray: July. In red: August. In dark gray: September. In black: October. **(C)** Correlation of gene expression data from spleen samples of Atlantic salmon. *: the significant proportional relationship between two groups of data (*P < *0.05).

Results of gene expression showed that *ifnγ* did not show significant differences in fish from FarmA during the surveillance period (**Figure 5A**). On the contrary, the spleen samples exhibited a higher expression of *ifnγ* in July (4.27-fold) and August (3.70-fold) compared to June (1.30-fold) (**Figure 5B**) in samples from FarmB.

Regarding *mhcii*, the gene expression of this molecule was significantly increased in fish from FarmA in October (2.61-fold higher expression compared to June: 1.73-fold), while in FarmB, *mhcii* expression showed a significant increase in August (2.12-fold) compared to June (1.39-fold).

No significant variation on gene expression was found for *cd83* or *cd80/86* in fish from FarmA. However, *cd83* expression showed a significant increase in July (1.37-fold), August (1.58-fold) and September (1.33-fold) compared to June (0.94-fold) in FarmB. Moreover, the expression of *cd80/86* in fish from FarmB showed a significant increase in August (2.07-fold), compared to June, July and October (1.38-fold, 1.65-fold, 1.49-fold, respectively), and in September (1.73-fold) compared to June (1.38-fold).

The correlation of gene expression data during the field surveillance trial showed a significant positive correlation of *cd80/86* with *mhcii* in FarmA (**Figure 5C**). In addition, significant positive correlations were found between: *ifnγ* and *cd83*, *cd83* and *cd80/86*, *cd83* and *mhcii*, *cd80/86* and *mhcii* in FarmB.

## Discussion

Fish are the most ancient and numerous groups of vertebrates since their explosive radiation in the Devonic period. This heterogeneous group of animals lays in the crossroads between the innate response and the appearance of the adaptive immunity (31). Fish have an immune system with physical, cellular, and humoral components that act in a coordinated manner for the recognition of the non-self and fight against foreign agents (32). In this scenario, a pleiotropic cytokine as IFNγ can regulate both the innate and adaptive immune response of animals (5).

In higher vertebrates, IFNγ is produced primarily by NK cells and CD4^+^ Th1 cells (3) and it is crucial to promoting Th1 responses (2), increasing the expression of cell-surface markers on dendritic cells, modulating the antigen presentation process and T cells polarization (3, 4).

In fish, IFNγ has been reported in several teleost families (5) and it can modulates the expression of proinflammatory cytokines and chemokines such as IL-1β, IL-6, IL-12, TNFα, CXCL9/10/11 (6) and IDO (Indoleamine-pyrrole 2,3-dioxygenase), which is related with metabolic immune regulation (19). Moreover, IFNγ strongly enhances the phagocytic and nitric oxide responses of phagocytes in fish (5).

Following the method proposed by Bassity and Clark (18), which describes the protocol to stimulate, obtain and identify dendritic cells-like (DC-like) in teleost models, the present work was able to detect that a small population of mononuclear splenocytes phenotypically progress to a larger cell size morphology. These findings were consistent with those reported by Haugland et al. (33), who suggested that small mononuclear blood cells from salmon, with high phagocytic capacity, have the ability to differentiate into DC-like.

We also were able to establish that after two weeks of stimulation with IFNγ, salmon splenocytes were able to increase cell-surface markers such as CD80/86, CD83 and MHC II and decrease the expression of zbtb46, all molecules associated with APCs. These results suggest a differentiation of splenocytes towards activate DC-like, since CD83 is a marker for mature DCs in higher vertebrates (13, 34).

In mammals, IFNγ can upregulate to CD83 in peripheral blood mononuclear cells (PBMC), modulating the immune response, activating dendritic cells and delivering co-stimulatory signals for the activation of human naive T cells (13). Furthermore, in mice, IFNγ increases the expression of *cd80* and *cd86* and *mhcii* during the antigen presentation processes (4, 35).

Regarding fish, IFNγ induces MHC II expression mediated by Class II Transactivator (CIITA) in grass carp (7). Furthermore, Wang et al. (8) described that the stimulation with IFNγ can produce a higher level of MHC-I and MHC-II in trout macrophages. The same authors propose that this would be related to the inhibition of zbtb46 expression, which is a transcriptional factor that keeps arrested the phenotypic progression of APCs in higher vertebrates.

The results obtained *in vitro* led to the need to evaluate antigen-presenting cells markers at *in vivo* level. To do this, we monitored the expression of *ifnγ*, *cd80*/*86*, *cd83* and *mhcii* for 5 months in two fish farms, considering the abiotic and biological conditions that the environment presented during the *in vivo* approach. Interestingly, only fish from FarmB, which reported an outbreak of *P. salmonis* in August, presented an increasing level of *ifnγ* (both before and during the outbreak), with simultaneous higher expression of *cd80/86*, *cd83* and *mhcii* in the spleen of Atlantic salmon. Furthermore, when the proportionality of the gene expression from the different markers was determined, we could establish that *ifnγ* in FarmB was positively correlated to the others APCs markers. On the contrary, in FarmA, a positive correlation was only observed between *cd80/86* and *mhcii*, but this was not related to the expression of *ifnγ* and *cd83*. This may be due to the stimulation of a response from the host that seeks to control the infection of an intracellular pathogen during the outbreak of *P. salmonis* (in FarmB).

It has already been reported that *P. salmonis* infection can provoke an IFN-inducible response (36). Our *in vitro* results could be related to these field data, since an increase of IFNγ may stimulate the expression of cell-surface markers in APCs to trigger an enhanced immune response in mammals (3, 4, 35).

In summary, the stimulation of splenocytes with IFNγ for 15 days down-regulates the expression of *zbtb46*, and induces a higher level of CD80/86, CD83 and MHCII. The increase in the expression of cell-surface markers was also observed under field conditions when *ifnγ* was upregulated. We hypothesize that some of the problems associated with vaccines in fish, which resulting in low protection over time, may be due to the low number of APCs with a mature phenotype. Finally, we propose the use of IFNγ in immuno-modulatory strategies that stimulate the phenotypic progression of immune cells. Further studies on the effects of IFNγ must be performed *in vivo*, under-productive conditions, and with different concentrations and kinetics to achieve such task.

## Data Availability Statement

The datasets presented in this study can be found in online repositories. The names of the repository/repositories and accession number(s) can be found in the article/**Supplementary Material**.

## Ethics Statement

The animal study was reviewed and approved by Comité de Bioética Pontificia Universidad Católica de Valparaíso.

## Author Contributions

The study was conceived by BM-L and LM with extensive additional input from PS, CS, JA, MI, LL, MØ, and SB. FG produced the synthetic peptides. The experiments and data analysis were performed by BM-L, FR, VW-B, DF, and LL. The funds for this investigation were acquired by LM, LL, CS, and MØ. PS drafted the manuscript with substantial contributions from all other authors. All authors contributed to the article and approved the submitted version.

## Funding

This work was supported by the Program for Sanitary Management in Aquaculture of the Ministry of Economy, Development and Tourism of Chile (SERNAPESCA FIE-2015-V014 201708070149), National Fund for Scientific and Technological Development (FONDECYT 1191763), and the Nutrition and Immunity project (281052) of The Research Council of Norway.

## Conflict of Interest

JA was employed by the company AquaAdvise. CS was employed by the company Salmones Camanchaca.

The remaining authors declare that the research was conducted in the absence of any commercial or financial relationships that could be construed as a potential conflict of interest.
