# Supplementary figures and images for: Interferon Gamma Induces the Increase of Cell-Surface Markers (CD80/86, CD83 and MHC-II) in Splenocytes From Atlantic Salmon
Source: Front Immunol. 2021 May 13;12:666356. doi: 10.3389/fimmu.2021.666356 (PMC8155612; doi:10.3389/fimmu.2021.666356)

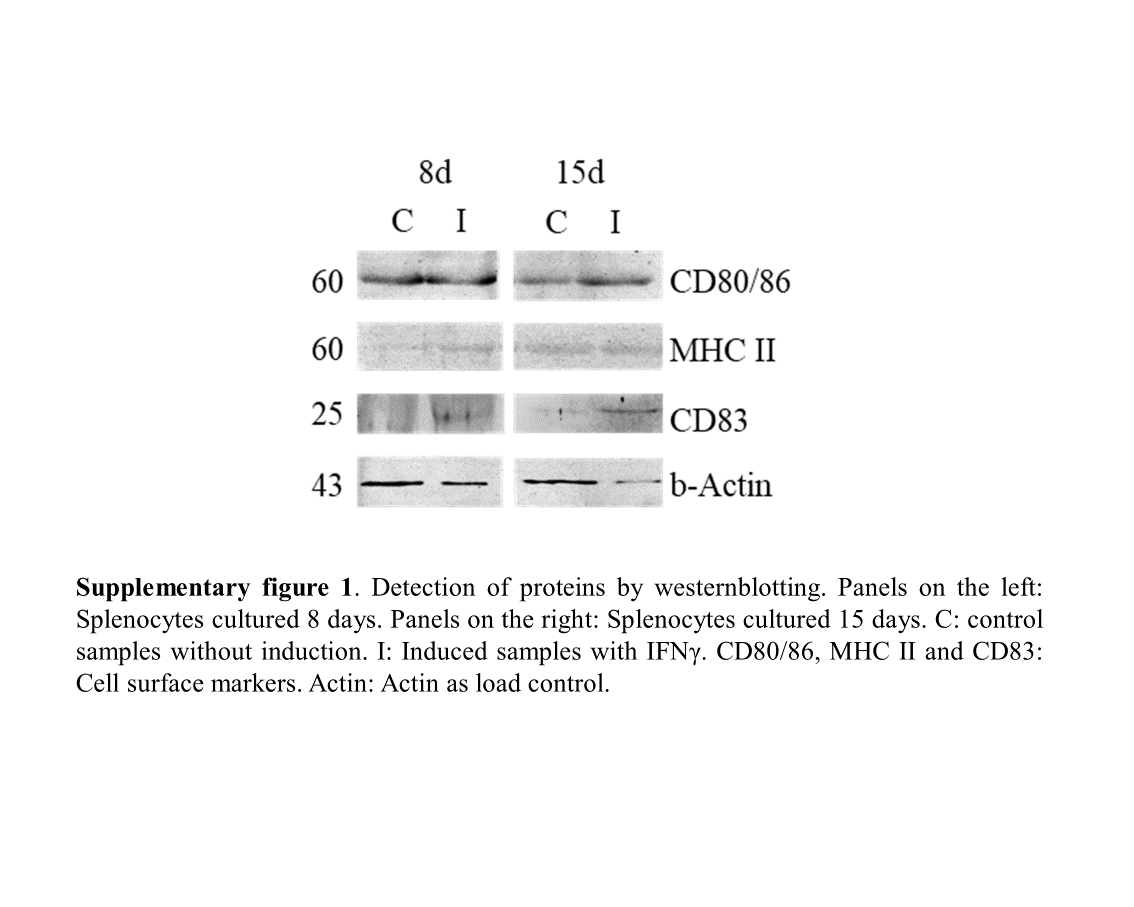

Supplement: Supplementary file 1 [file Image_1.tif]
